# Malus-metasurface-assisted polarization multiplexing

**DOI:** 10.1038/s41377-020-0327-7

**Published:** 2020-06-12

**Authors:** Liangui Deng, Juan Deng, Zhiqiang Guan, Jin Tao, Yang Chen, Yan Yang, Daxiao Zhang, Jibo Tang, Zhongyang Li, Zile Li, Shaohua Yu, Guoxing Zheng, Hongxing Xu, Cheng-Wei Qiu, Shuang Zhang

**Affiliations:** 1 Electronic Information School, Wuhan University, 430072 Wuhan, China; 2 NOEIC, State Key Laboratory of Optical Communication Technologies and Networks, Wuhan Research Institute of Posts & Telecommunications, 430074 Wuhan, China; 3 School of Physics and Technology, Wuhan University, 430072 Wuhan, China; 4 Department of Electrical and Computer Engineering, National University of Singapore, 4 Engineering Drive 3, Singapore, 117583 Singapore; 5 Integrated Circuit Advanced Process Center, Institute of Microelectronics, Chinese Academy of Sciences, 100029 Beijing, China; 6 School of Physics & Astronomy, University of Birmingham, Birmingham, B15 2TT UK

**Keywords:** Nanophotonics and plasmonics, Metamaterials

## Abstract

Polarization optics plays a pivotal role in diffractive, refractive, and emerging flat optics, and has been widely employed in contemporary optical industries and daily life. Advanced polarization manipulation leads to robust control of the polarization direction of light. Nevertheless, polarization control has been studied largely independent of the phase or intensity of light. Here, we propose and experimentally validate a Malus-metasurface-assisted paradigm to enable simultaneous and independent control of the intensity and phase properties of light simply by polarization modulation. The orientation degeneracy of the classical Malus’s law implies a new degree of freedom and enables us to establish a one-to-many mapping strategy for designing anisotropic plasmonic nanostructures to engineer the Pancharatnam–Berry phase profile, while keeping the continuous intensity modulation unchanged. The proposed Malus metadevice can thus generate a near-field greyscale pattern, and project an independent far-field holographic image using an ultrathin and single-sized metasurface. This concept opens up distinct dimensions for conventional polarization optics, which allows one to merge the functionality of phase manipulation into an amplitude-manipulation-assisted optical component to form a multifunctional nano-optical device without increasing the complexity of the nanostructures. It can empower advanced applications in information multiplexing and encryption, anti-counterfeiting, dual-channel display for virtual/augmented reality, and many other related fields.

## Metasurfaces: nanobrick polarizers

Metasurfaces made from an array of carefully orientated silver nano-brick polarizers on a silica glass substrate can manipulate the phase and amplitude of light. Fabricated and characterized by a team of researchers in China, Singapore and the UK, the so-called Malus metasurfaces are optimally designed to operate with red light and generate near-field greyscale patterns and far-field holographic images. Potential uses include image multiplexing and encryption, augmented-reality displays and other applications. The metasurfaces consist of nanoscale (160 nm long, 80 nm wide and 70 nm high) cuboids of silver which have a long axis and short axis. Simulations indicate that the nanobricks reflect 92.6% of incident 633 nm-light when its polarization is aligned along the long axis and transmit 95.3% when aligned to its short axis, thus acting as a nanoscale polarizer.

## Introduction

As three fundamental properties of light, the polarization, amplitude, and phase are naturally independent and interrelated. Malus’s law, a basic principle in polarization optics, is governed by the simple mathematical equation *I* = *I*_0_ cos^2^*θ*, which describes the intensity of a linearly polarized (LP) beam passing through a polarizer. In the equation, the mapping between the output intensity *I* and the polarization angle *θ* indicates that the intensity (or amplitude) and polarization of light can be simultaneously controlled by Malus devices, such as liquid crystal displays and polarized filters.

Degeneracy, a widely used concept in biology, medicine, quantum mechanics, and mathematics, is employed to describe multiple states under the same mapping result. Due to the orientation degeneracy implied in Malus’s law, we note that there exists a one-to-*M* mapping (i.e., the degeneracy is *M*) between the light irradiance and orientation angle of a polarizer, which can be combined with the Pancharatnam–Berry (PB) phase^1,2^ and applied in designing advanced geometric metasurfaces (GEMSs). GEMSs^2–13^, artificially engineered planar metastructures, have emerged as a powerful means to manipulate the PB phase delay (exactly twice the nanostructure orientation angle) cell-by-cell under circularly polarized (CP) incident light, independent of the wavelength or specific nanostructure design.

On the basis of the abovementioned principle, we propose Malus metasurfaces capable of simultaneously and independently manipulating the intensity and phase of light simply by polarization modulation. Specifically, we can design each of the nanostructure orientations to achieve different PB phase manipulations while keeping the intensity manipulation unchanged. Therefore, a metasurface consisting of an array of *N* nanostructures has *M*^*N*^ different combinations of orientations that can generate the same intensity profile of incident LP light but different geometric phase distributions for incident CP light. Therefore, Malus metasurfaces can act as both intensity modulators and phase modulators simply by manipulating the polarization of incident light.

Recently, polarization, amplitude, and phase modulations based on metasurfaces have been extensively studied for information multiplexing^14–39^. The advanced anisotropy manipulation of metasurfaces indicates the capability of achieving dual polarization multiplexing for two beams of orthogonal LP light. For example, by changing the arm length of a cross-shaped nanostructure^18,19^ or the dimensions of a nanoscale pillar^20–24^, the phase or amplitude of orthogonal LP light can be independently controlled, and then, polarizing beam splitters^18,20^, dual-channel nanoprinting devices^19,21,22^, step-zoom lenses^23^, 3D holograms^24^, and devices with other functionalities can be realized. Furthermore, the phase of orthogonal CP light^25^, any orthogonal polarized light^26^, or even nonorthogonal polarized light^27^ can be independently modulated in two polarization modes by simultaneously elaborately designing the dimensions and orientations of nanostructures. Metasurfaces can also achieve independent phase control in multiple polarization modes^28–30^. The above multiplexed metasurfaces have been applied in many fields, such as multichannel holograms^28^, color holograms^29^, and polarization cameras^30^. In addition, a variety of schemes have been proposed to simultaneously realize amplitude and phase control (i.e., complex amplitude modulation), implemented by utilizing the coupling effect between two nanostructures^31^, changing the geometry dimensions of nanostructures to form a set of wave plates with varied phase difference^32^, interleaving multiple metasurfaces in plane^33–36^, stacking metasurfaces in space^37–39^, etc. However, both the polarization multiplexing and complex amplitude modulation mentioned above require complex nanostructure design or sacrifice of some control of the optical transmission matrix, which therefore burdens the fabrication process or decreases the information density of each image channel.

In comparison, our approach provides a new degree of freedom to design multiplexed metasurfaces and allows one to manipulate both the amplitude and phase of light independently. More importantly, our strategy does not increase the complexity of the nanostructures or decrease the image quality and can thus pave a new way for developing high-density and multifunctional metasurfaces with an ultrasimple approach. Specifically, we theoretically and experimentally demonstrate a Malus-metasurface-assisted paradigm by realizing two independent information channels with a single-sized metasurface for incident LP and CP light, which simultaneously presents a high-resolution continuous greyscale pattern at the sample surface and a multistep phase-only holographic image in the far field. The proposed Malus metasurfaces, featuring complete independence between amplitude and phase manipulation and spatially separated information in the near and far fields, can find various applications in information multiplexing, high-end optical anti-counterfeiting, information hiding and encoding, planar lightwave circuitry, dual-channel display for AR/VR, optical storage and many other related fields.

## Results

### One-to-two mapping scheme

We start by considering a simple case with a one-to-two mapping between the intensity of light and the orientation of each nanostructure. When incident LP light (the polarization direction is *α*_1_) with an intensity of *I*_0_ passes through an anisotropic nanostructure with an in-plane orientation *θ*, the intensity of the transmitted light can be derived as

1 $$I_1 = I_0\left[ {A^2\cos ^2(\theta - \alpha _1) + B^2\sin ^2(\theta - \alpha _1)} \right]$$

where *A* and *B* are the complex transmission coefficients for incident light LP along the long and short axes of the nanobrick, respectively. (See Section 1 of the Supplementary materials for details of the equation derivation.) Specifically, when the nanostructure acts as an ideal polarizer (i.e., *A* = 0 and *B* = 1), we can simplify the intensity of the transmitted light as

2 $$I_1 = I_0{\mathrm{sin}}^2(\theta - \alpha _1)$$

As a result, for incident light polarized along a given direction (assuming *α*_1_ is fixing), a continuous greyscale pattern can be formed at the surface by configuring the orientation angles *θ* of the nano-polarizer array. Meanwhile, in the defined orientation angle interval [0, *π*], each nano-polarizer has two orientation options to produce the same output intensity, which can be used to form a two-step GEMS. More details about the expressions of the two different orientation angles are summarized in Table S1 of the Supplementary materials.

### One-to-four mapping scheme

A two-step GEMS cannot avoid the issue of generating twin images in principle, and the diffraction efficiency is only 40.5% theoretically^40^. To increase the quantization steps of GEMSs, we propose a one-to-four mapping scheme: only by simply inserting a bulk analyser into the same optical setup can a four-step GEMS be attained while maintaining the other conditions unchanged. Specifically, when incident LP light successively passes through a nano-polarizer and a bulk analyser, the intensity of the transmitted light can be expressed as

3 $$I_2 = I_0\left[ {\frac{{A - B}}{2}\cos \left( {2\theta - \alpha _2 - \alpha _1} \right) + \frac{{A + B}}{2}\cos \left( {\alpha _2 - \alpha _1} \right)} \right]^2$$

where *α*_2_ represents the direction of the bulk analyser transmission axis. In particular, by setting *α*_2_*α*_1_ + *π*/2, Eq. (3) can be rewritten as

4 $$I_2 = I_0\left( {\frac{{A - B}}{2}} \right)^2\cos ^2\left( {2\theta - 2\alpha _1 - {\pi}/2} \right)$$

From Eq. (4), one finds that there are four orientation angles in the defined interval [0, *π*] that can produce the same output intensity (more details about the expressions of the four different orientation angles are summarized in Table S1 of the Supplementary materials). This provides the capability for metasurfaces to manipulate the PB phase under the illumination of CP light. Therefore, on the basis of the one-to-four mapping scheme, we can employ not only continuous intensity manipulation but also four-step phase manipulation without complicating the design and fabrication of the nanostructure.

### Demonstration of Malus metasurfaces based on one-to-two mapping

**Fig. 1:**
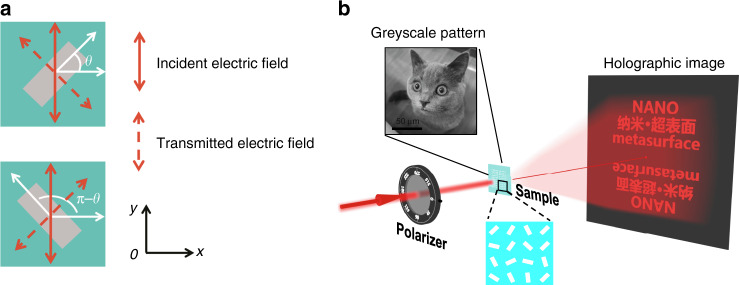
Schematic of Malus metasurfaces based on one-to-two mapping and two independent information channels for intensity and phase manipulation. **a** Each nanobrick polarizer has two orientation angle options to produce the same transmitted intensity but different geometric phase delays. Here, *α*_1_ = *π*/2. **b** A continuous greyscale pattern with intensity manipulation is recorded on the metasurface under incident LP light (observed with an optical microscope), while a Fourier holographic image with phase manipulation is reconstructed in the far field under laser source illumination (observed with the naked eye or a camera).

According to Eq. (2), when LP light with polarization orientation angle *α*_1_ = *π*/2 passes through a metasurface, the corresponding intensity of transmitted light can be simplified as *I*_1_ = *I*_0_ cos^2^*θ*, which indicates that each nano-polarizer with orientation angle *θ* or *π* − *θ* can produce equal intensity of transmitted light but different phase delays (Fig. 1a). The freedom provided by the one-to-two mapping scheme allows us to record a continuous greyscale pattern (intensity manipulation) on the nanostructure surface and simultaneously produce a two-step phase-only holographic image (phase manipulation) in the far field (Fig. 1b). Here, we utilize silver nanobrick arrays sitting on a glass substrate to form the Malus metasurface. By elaborately designing the geometric size, each silver nanobrick can function as a nano-polarizer and transmit the incident beam polarized along the nanobrick short axis while reflecting that polarized along the long axis.

Based on the above designed nanobrick, we encode two independent target patterns (one for the greyscale pattern and another for the holographic image) into a single metasurface. First, according to the greyscale distribution of the target pattern, we calculate all possible combinations of nanobrick orientation angles. Since each pixel cell has two options for the nanobrick orientation, a simulated annealing algorithm^41^ is applied to optimize the orientation distribution (more details are presented in Fig. S1), and then, the Fourier GEMS hologram is formed (the phase is exactly twice the orientation angle).

**Fig. 2:**
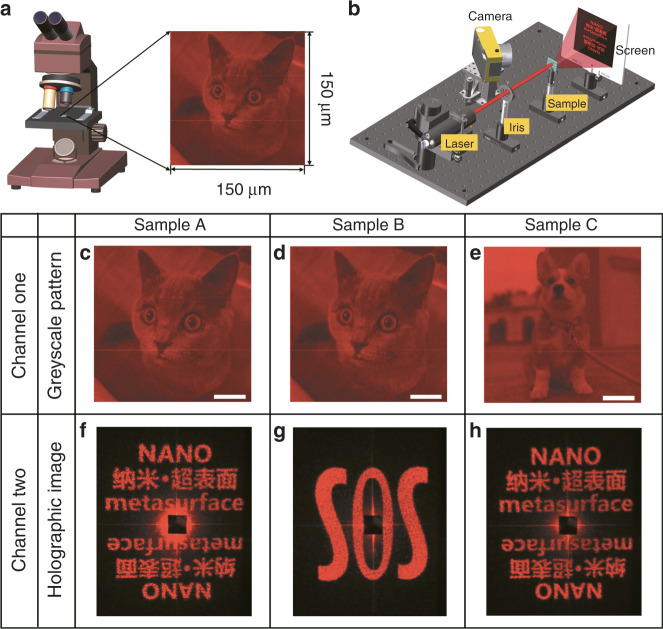
Experimental setup and results of the Malus metasurfaces based on one-to-two mapping. **a** An optical microscope (Motic BA310Met) is used to capture the greyscale pattern when LP light illuminates the sample. **b** The transmitted holographic image can be generated in the far field when an LP light beam emitted by a supercontinuum laser illuminates the metasurface sample. Second row **c–e**: experimental greyscale patterns under the illumination of LP light (the operating wavelength is 633 nm). Third row **f–h**: experimental Fourier holographic images under LP light illumination. Samples A and B can generate the same greyscale pattern of a “cat” in the near field but different holographic images in the far field. Samples A and C can generate different greyscale patterns of a “cat” and a “dog” in the near field but the same holographic image of English letters and Chinese characters in the far field. Samples A–C are designed with dimensions of 150 × 150 μm^2^. The scale bar is 30 μm. The holographic images of samples A–C are designed to create wide image angles of 59° × 66°, 50° × 50°, and 59° × 66°, respectively.

To demonstrate the feasibility and flexibility of the Malus metasurfaces based on one-to-two mapping, three different samples (labeled A–C) were fabricated with electron beam lithography. Figure S2 shows scanning electron microscopy (SEM) images of a partial region of one metasurface sample. For samples A and B, we encode the same near-field greyscale pattern but different far-field holographic images. Samples A and C are designed to generate the same far-field holographic image but different near-field greyscale patterns. Figure 2a, b shows the experimental setup used for characterizing the metasurfaces. As a result, a greyscale pattern of a “cat” with high resolution and fidelity can be observed by an optical microscope (the central wavelength is 633 nm) under LP light illumination. In another measurement for the same sample, when sample A is normally illuminated by a laser source, a holographic image containing English letters and Chinese characters appears in the far field (300 mm from the sample). Sample B shows the same greyscale pattern as sample A but a totally different holographic image (letters of “SOS” with a centrosymmetric design), as shown in Fig. 2d, g. Meanwhile, sample C shows a different near-field pattern of a “dog” but the same far-field holographic image as sample A, as shown in Fig. 2e, h. All results are in good accordance with our design.

### Demonstration of Malus metasurfaces based on one-to-four mapping

**Fig. 3:**
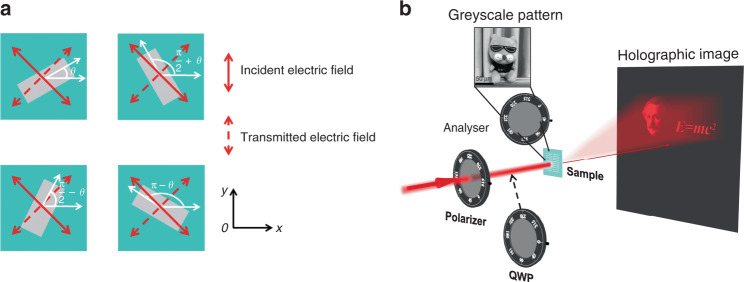
Schematic of Malus metasurfaces based on one-to-four mapping and two independent information channels for intensity and phase manipulation. **a** Each nanobrick polarizer has four orientation angle options to produce equal transmitted beam intensity but different geometric phase delays. Here, *α*_1_ = −*π*/4, and *α*_2_ = *π*/4. **b** Under LP light illumination, a continuous greyscale pattern with intensity manipulation can be encoded at the sample surface. When a QWP is inserted into the optical path to convert incident LP light into CP light, a four-step holographic image with phase manipulation can be reconstructed in the far field.

A four-step metasurface can be designed to project complex holographic images while eliminating the inevitable “twin-image” problem of two-step metasurfaces. For the one-to-four mapping (Eq. (4)), if *α*_2_ = *α*_1_ + *π*/2 = *π*/4, that is, an LP beam with polarization orientation angle *α*_1_ = −*π*/4 passes through a nanobrick polarizer and then an analyser with polarization orientation angle *α*_2_ = *π*/4, the simplified formula $I_2 = \frac{{I_0}}{4}\cos ^2\left( {2\theta } \right)$ can be utilized for describing the intensity of the transmitted light. As a result, there are four orientation angles of *θ*, *π*/2 + *θ*, *π*/2 − *θ*, and *π* − *θ* that can produce equal transmitted beam intensity but different PB phase delays of 2*θ*, *π* + 2*θ*, *π* − 2*θ*, and 2*π* − 2*θ*, as shown in Fig. 3a. Therefore, we can obtain a continuous intensity manipulation as well as a four-step phase manipulation with only one metasurface.

**Fig. 4:**
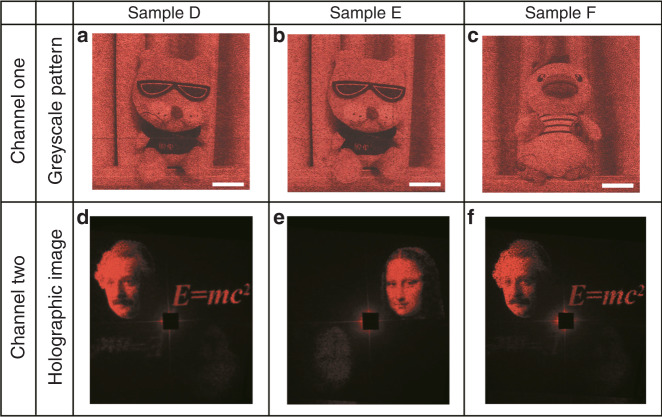
Experimental results of the Malus metasurfaces based on one-to-four mapping. First row **a–c**: experimental greyscale patterns under the illumination of LP light when the operating wavelength is 633 nm. Second row **d–f**: experimental holographic images under CP light illumination. Samples D and E can generate the same greyscale pattern (“dog doll”) in the near field but different holographic images (Einstein and Mona Lisa’s portraits, respectively) in the far field. Samples D and F can generate different greyscale patterns of a “dog doll” and a “duck doll” but the same holographic image of Einstein’s portrait. Samples D–F are designed with dimensions of 150 × 150 μm^2^. The scale bar is 30 μm. The holographic images of samples D–F are designed to create wide image angles of 85° × 41°, 34° × 38°, and 85° × 41°, respectively.

With the same nanostructural parameters and Fourier hologram design algorithm, we further design and fabricate three different samples (labeled D–F). The arrangement of the three samples is the same as that of samples A–C, i.e., samples D and E have the same greyscale pattern but different holographic images, whereas samples D and F have the same holographic image but different greyscale patterns. As shown by the experimental results in Fig. 4, sample D can not only encode a high-resolution greyscale pattern of a “dog doll” in the near field but also generate a holographic image with high fidelity (Einstein’s portrait) in the far field, which indicates the feasibility of Malus metasurfaces based on one-to-four mapping. More interestingly, the twin-image that the two-step hologram always suffers from disappears (only very weak residual images exist because of algorithm residuals and sample and polarizer fabrication errors), as shown in Fig. 4d–f. For samples E and F, the experimental results have good consistency with our design, as shown in Fig. 4b, e, c, f. In summary, all experimental results show that the two information channels are independent, so we can design target patterns for meta-nanoprinting and meta-holography at will.

The measured hologram efficiency, defined as the ratio of the power of the transmitted holographic image to the power of the incident beam, is 7% at the operating wavelength of 633 nm. The efficiency could be improved by applying more precise fabrication procedures, using low-loss dielectric materials (such as TiO_2_) or reducing the coverage angles of the holographic image. More details about the efficiency calculation and measurement are discussed in Figs. S3 and S4 of the Supplementary materials.

The proposed Malus metasurfaces have great technical advantages. Generally, most optical devices can only work in either reflection or transmission. A significant feature of the Malus metasurfaces is that they can work not only in transmission but also in reflection, which greatly facilitates practical applications since the working modes can be chosen at will. Supplementary Fig. S5 shows all experimental results of samples A–F working in reflection. Compared with their transmission counterparts, the contrast of the reflection greyscale patterns has a slight reduction. The reason is that the unwanted reflectivity *Ry* is slightly higher than the unwanted transmissivity *T*_*x*_ when the operating wavelength is 633 nm (Fig. 6b shows the numerical simulation results). Meanwhile, reflective far-field holographic images with high fidelity can be observed, as shown in Fig. S5d–f, j–l.

To explore the broadband response characteristics of the Malus metasurfaces in the near field, we acquired the greyscale patterns under illumination by a quartz halogen lamp using an optical microscope, as shown in Supplementary Fig. S6. The greyscale patterns obtained in reflection and transmission show very clear visual effects. Then, to explore the spectral response in the far field, we utilized a supercontinuum laser (the wavelength varied within the range of 480–680 nm) to illuminate samples A and D. From Supplementary Fig. S7, one can see that all the holographic images possess high fidelity in both transmission and reflection. Benefiting from the broadband response characteristics of the metadevice we propose, the observation requirements in practical applications will be greatly reduced.

It is noted that the proposed one-to-four mapping scheme does not require the nanostructure to act as a perfect polarizer or a half-wave plate; that is, this scheme can be realized by using any nanostructure with anisotropy (*A* ≠ *B*). According to Eq. (4), when the polarization directions of normally incident LP light and the bulk analyser are orthogonal to each other (i.e., *α*_2_ = *α*_1_ + *π*/2), any desired intensity of the transmitted beam can be achieved by arranging the orientation angles of the anisotropic nanostructures. More details about the general concept of the one-to-*M* mapping scheme are discussed in Section 1 of the Supplementary materials. This characteristic is significant because a large fabrication error of metasurfaces is acceptable in principle, which may greatly improve the fabrication tolerance for mass production in industrial applications.

### Characteristics of pattern hiding based on polarization manipulation

**Fig. 5:**
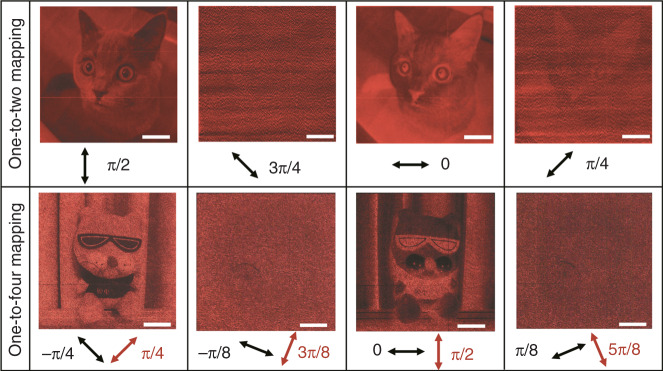
Experimental results for greyscale patterns with various polarization directions of incident LP light and the analyser. The first row shows the experimental results of sample A under the illumination of LP light with polarization orientation angles of *π*/2, 3*π*/4, 0, and *π*/4. The second row presents the results of sample D for LP light with polarization orientation angles of −*π*/4, −*π*/8, 0, and *π*/8, and the analyser has corresponding polarization orientation angles of *π*/4, 3*π*/8, *π*/2, and 5*π*/8, respectively. The black and red arrows represent the polarization orientation angles of the normally incident LP light and the analyser, respectively. The scale bar is 30 μm.

The proposed one-to-*M* mapping strategy destroys the overall consistency between the greyscale pattern and the polarization orientation angle of incident light. On the other hand, we can benefit from this characteristic to increase the security of the greyscale pattern. For a Malus metasurface based on one-to-two mapping (sample A), we rotate the polarization orientation angle of incident LP light from the designed *π*/2 to 3*π*/4, 0 and *π*/4 and present the experimental results in the first row of Fig. 5. When the polarization orientation angle is zero degrees, the brightness of the greyscale pattern is complementary to the result at *π*/2, and the result can be easily interpreted by Malus’s law. It is interesting that when the polarization orientation angles are *π*/4 and 3*π*/4, the greyscale patterns are almost lost in the background noise. For the metasurfaces based on the one-to-four mapping design, a similar phenomenon is observed (shown in the second row of Fig. 5).

The destruction of the greyscale patterns can be explained by observing the mathematical functions cos^2^*θ* and cos^2^2*θ*, which determine the intensity of transmitted light. When the polarization orientation angles of the incident light have deviation values of *π*/4 and *π*/8, respectively, the intensity of transmitted light is proportional to cos^2^(*θ* − *π*/4) and cos^2^(2*θ* − *π*/8), respectively. Since each orientation angle *θ* has two or four “random” options depending on the geometric phase design, the intensity of the transmitted light will become rather irregular, which leads to destruction of the greyscale pattern. Such a design is quite different from conventional one-to-one mapping, in which LP light with any polarization orientation angle can form different but recognizable greyscale patterns^42–45^. As a comparison, we simulate greyscale patterns with one-to-one, one-to-two, and one-to-four mapping designs, and the results are shown in Supplementary Fig. S8. Notably, this distinctive polarization-dependent characteristic can be used to increase the security of greyscale patterns and make them difficult to decode and duplicate.

## Discussion

One direct application of the proposed Malus metasurfaces is information multiplexing, which can provide a completely new information channel with phase manipulation in the far field in addition to the information channel with amplitude manipulation at the nanostructure surface. We note that there have been recent reports on the combination of nanoprinting and holography^13,31–39^; however, our concept does not conflict with their schemes, and it provides an extra degree of freedom to further increase the information capacity. Furthermore, it is promising to merge wavelength multiplexing^46^ with the proposed Malus metasurfaces to simultaneously generate a near-field colorful pattern and far-field holographic images. The propagation phase^47–49^ can also be merged into the design of Malus metasurfaces to further extend their functionalities. In addition, our proposed scheme is not limited to one-to-four mapping, and it can be further extended to one-to-eight mapping by stacking bilayer nanostructures in space (more details are presented in Section 8 of the Supplementary materials).

In addition to information multiplexing, we also propose another potential application of optical anti-counterfeiting with multiple verification rules based on metasurfaces. Our Malus metasurfaces can record independent complex information in both the near and far fields, which provides two different security verification rules. In addition, benefiting from the polarization-dependent characteristics of the metadevice, the near-field complicated information can only be observed when the incident light is polarized along the given direction, corresponding to a third security verification rule. Therefore, compared with conventional techniques, such as holography, our proposed multifunctional metadevice can make optical anti-counterfeiting more secure. Malus metasurfaces are also promising in VR/AR displays by coating the surface of an intelligent glass or eyepiece with a Malus metasurface. As a result, if the observer focuses on the surface (near field), greyscale patterns can be captured; if he or she focuses on infinity (far field), he or she can observe the holographic images being blended with real world scenes.

Finally, the proposed method is not limited to nanostructures, and all conventional polarization devices obeying Malus’s law can have an extra degree of freedom for phase manipulation. For example, optically patterned retarders composed of linearly polymerized liquid crystal aligning layers^50^ can be employed to form multifunctional devices suitable for large-area and mass fabrication. The combination of orientation degeneracy and the PB phase provides great flexibility in the design of multifunctional nano-optical devices functioning as both intensity modulators and phase modulators, which has major implications for information optics and integrated optics. It is expected that a variety of novel nano-optical devices applied in information multiplexing, high-end multifold anti-counterfeiting, information encoding and hiding, planar lightwave circuitry, high-density optical storage, dual-channel display for AR/VR, and many other related applications will emerge from our approach.

In summary, inspired by the orientation degeneracy of the mathematical function cos^2^*θ* in Malus’s law, we propose a one-to-*M* mapping scheme for advanced Malus metasurface design, which breaks the conventional one-to-one mapping strategy between the intensity of output light and the orientation of nanostructures, providing an extra degree of freedom for geometric phase design. We have designed two types of Malus metasurfaces that generate continuous greyscale patterns encoded at the sample surface while projecting two-step or four-step phase-only holographic images in the far field. The experimental results indicate that the phase manipulation functionality can be merged into an amplitude-manipulation-assisted nano-optical component to form a multifunctional nano-optical device without increasing the complexity of the nanostructures. The independent control of the intensity and phase, multifunctionality, compatibility, broadband working window, simple structure, and robustness against fabrication errors endow Malus metasurfaces with great potential in various applications and promote the emergence of a variety of novel nano-optical elements and devices.

## Materials and methods

### Design of a unit cell of the Malus metasurface

**Fig. 6:**
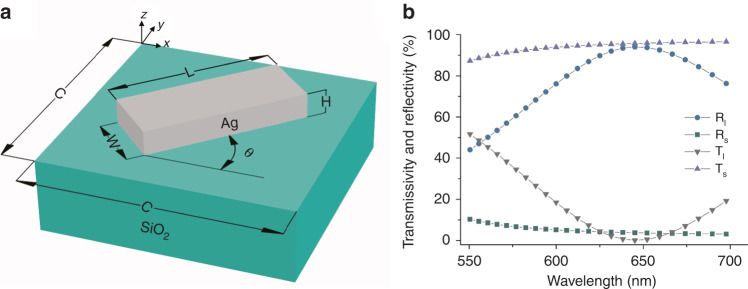
Schematic illustration of a nanobrick-based polarizer. **a** Schematic illustration of a Ag nanobrick. **b** Simulated results (*R*_l_, *R*_s_, *T*_l_, *T*_s_) versus incident wavelength.

Figure 6a shows a schematic illustration of a Ag nanobrick positioned on a glass substrate. To ensure that the beam with polarization direction along the nanobrick short axis will be totally transmitted while the orthogonal polarization will be totally reflected at the design wavelength of 633 nm, we carry out numerical simulations by utilizing CST Microwave Studio software. As a result, the cell size, length, width, and height of the optimized nanobrick polarizer are 300, 160, 80, and 70 nm, respectively. Figure 6b shows the simulated reflectivity (*R*_s_, *R*_l_) and transmissivity (*T*_s_, *T*_l_) when the polarization direction of the incident beam is along the short and long axes of the nanobrick. We found that the desired reflectivity *R*_l_ and transmissivity *T*_s_ reach 92.6% and 95.3% at the design wavelength of 633 nm, and the unwanted *R*_s_ and *T*_l_ can be suppressed to below 4% and 2%. Therefore, the nanobrick can act as a nano-polarizer in both transmission and reflection.

### Fabrication of silver nanobrick polarizer arrays

We fabricated the sample on a piece of SiO_2_ substrate by electron beam lithography (EBL). First, to generate a conducting layer, a PMMA film was spin coated and covered by a PEDOT:PSS film. Then, we used electron beam lithography (Raith eLINE Plus, 30 kV) to expose the pattern of the nanobrick array. The conducting layer was washed away after being exposed, and the electron beam resist was developed at 22 °C for 80 s. After that, the sample was immediately washed with IPA for 30 s and dried by nitrogen. Next, 3 nm titanium and 70 nm silver were successively deposited by an electron beam evaporator. Finally, the desired metasurface structure was obtained by using hot acetone to clean off the excess metal film and the electron beam resist.

## Supplementary information

Supplementary information for Malus-metasurface-assisted polarization multiplexing
