# Supplementary material for: Malus-metasurface-assisted polarization multiplexing
Source: Light Sci Appl. 2020 Jun 12;9:101. doi: 10.1038/s41377-020-0327-7 (PMC7293268; doi:10.1038/s41377-020-0327-7)
Supplement: Supplementary file 1 — Supplementary information for Malus-metasurface-assisted polarization multiplexing [file 41377_2020_327_MOESM1_ESM.docx]

Supplementary information for

**Malus-metasurface-assisted polarization multiplexing**

**Liangui Deng^1,2,#^, Juan Deng^1,#^, Zhiqiang Guan^3,#^, Jin Tao^2,#^, Yang Chen^4^, Yan Yang^5^, Daxiao Zhang^3^, Jibo Tang^3^, Zhongyang Li^1^, Zile Li^1,*^, Shaohua Yu^2,*^, Guoxing Zheng^1,2,*^ Hongxing Xu^3,*^, Cheng-Wei Qiu^4^ and Shuang Zhang^6^**

^1^Electronic Information School, Wuhan University, Wuhan 430072, China

^2^NOEIC, State Key Laboratory of Optical Communication Technologies and Networks, Wuhan Research Institute of Posts & Telecommunications, Wuhan 430074, China

^3^School of Physics and Technology, Wuhan University, Wuhan 430072, China

^4^Department of Electrical and Computer Engineering, National University of Singapore, 4 Engineering Drive 3, Singapore, 117583, Singapore

^5^Integrated Circuit Advanced Process Center, Institute of Microelectronics, Chinese Academy of Sciences, Beijing 100029, China

^6^School of Physics & Astronomy, University of Birmingham, Birmingham B15 2TT, UK

^#^These authors contributed equally: Liangui Deng, Juan Deng, Zhiqiang Guan and Jin Tao

*Correspondence: Zile Li ([lizile@whu.edu.cn](mailto:lizile@whu.edu.cn)) or Shaohua Yu ([shuyu@wri.com.cn](mailto:shuyu@wri.com.cn)) or Guoxing Zheng ([gxzheng@whu.edu.cn](mailto:gxzheng@whu.edu.cn)) or Hongxing Xu ([hxxu@whu.edu.cn](mailto:hxxu@whu.edu.cn))

## 1. General concept of the one-to-many mapping scheme

The Jones matrix of an anisotropic nanobrick with an in-plane orientation *θ* can be expressed as

|  | $T\left( \theta\right)=R\left( -\theta\right)T_{0}R\left( \theta\right)=\left[ \begin{matrix} \cos\theta& -\sin\theta\\ \sin\theta& \cos\theta\end{matrix} \right]\left[ \begin{matrix} A & 0 \\ 0 & B \end{matrix} \right]\left[ \begin{matrix} \cos\theta& \sin\theta\\ -\sin\theta& \cos\theta\end{matrix} \right]$, | (S1) |
| --- | --- | --- |

where $R(\theta)$ is the rotate matrix, *A* and *B* are the complex transmission (or reflection) coefficients for incident linearly polarized (LP) light along the long and short axes of the nanobrick, respectively.

**1.1 One-to-two mapping scheme**

When an incident LP beam passes through a nanobrick, the Jones vector of transmitted beam can be expressed as

|  | $J_{1}=\left[ \begin{matrix} \cos\theta& -\sin\theta\\ \sin\theta& \cos\theta\end{matrix} \right]\left[ \begin{matrix} A & 0 \\ 0 & B \end{matrix} \right]\left[ \begin{matrix} \cos\theta& \sin\theta\\ -\sin\theta& \cos\theta\end{matrix} \right]\left[ \begin{aligned} {\cos\alpha}_{1} \\ {\sin\alpha}_{1} \end{aligned} \right]$  $=\left[ \begin{aligned} {A\cos(\theta-\alpha}_{1})\cos\theta+{Bsin(\theta-\alpha}_{1})sin\theta\\ {A\cos(\theta-\alpha}_{1})sin\theta-{Bsin(\theta-\alpha}_{1})cos\theta\end{aligned} \right]$, | (S2) |
| --- | --- | --- |

where $\alpha_{1}$ represents the polarization direction of the incident LP beam. If the intensity of incident LP beam is $I_{0}$, the intensity of transmitted beam can be expressed as

|  | $I_{1}=I_{0}\left[ A^{2}\cos^{2} \left( \theta-\alpha_{1} \right)+B^{2}\sin^{2} \left( \theta-\alpha_{1} \right) \right]$. | (S3) |
| --- | --- | --- |

Specifically, when the nanobrick acts as an ideal polarizer (i.e., $A=0$ and $B=1$), we can deduce the intensity of the transmitted beam as

|  | $I_{1}=I_{0}\sin^{2} \left( \theta-\alpha_{1} \right)$. | (S4) |
| --- | --- | --- |

From Eq. S4 we can see that any continuous greyscale can be acquired by rotating the polarization direction of a nanobrick based polarizer. Furthermore, when the incident beam is polarized at π/2, each nanobrick with orientations of $\cos^{-1} \sqrt{\frac{I_{1}}{I_{0}}}$ and ${-\cos}^{-1} \sqrt{\frac{I_{1}}{I_{0}}}$ can produce equal transmitted beam intensity due to the orientation degeneracy of the mathematical function $\cos^{2} \theta$.

**1.2 One-to-four mapping scheme**

When an incident LP beam passes through a nanobrick and an analyser, the Jones vector of transmitted beam can be expressed as

|  | $J_{2}=\left[ \begin{matrix} \cos^{2}\alpha_{2} & {\sin\alpha}_{2}{\cos\alpha}_{2} \\ {\sin\alpha}_{2}{\cos\alpha}_{2} & \sin^{2}\alpha_{2} \end{matrix} \right]\left[ \begin{matrix} \cos\theta& -\sin\theta\\ \sin\theta& \cos\theta\end{matrix} \right]\left[ \begin{matrix} A & 0 \\ 0 & B \end{matrix} \right]\left[ \begin{matrix} \cos\theta& \sin\theta\\ -\sin\theta& \cos\theta\end{matrix} \right]\left[ \begin{aligned} {\cos\alpha}_{1} \\ {\sin\alpha}_{1} \end{aligned} \right]$. | (S5) |
| --- | --- | --- |

If the intensity of incident LP beam is $I_{0}$, we can deduce the intensity of transmitted beam as

|  | $I_{2}={I_{0}\left[ \frac{A-B}{2}\cos\left( 2\theta-\alpha_{2}-\alpha_{1} \right)+\frac{A+B}{2}\cos\left( \alpha_{2}-\alpha_{1} \right) \right]}^{2}$, | (S6) |
| --- | --- | --- |

where *α*_1_ represents the polarization direction of an incident LP beam and *α*_2_ represents the polarization direction of the analyser.

**Case one:** if the nanobrick acts as an ideal polarizer (i.e. *A*=0 and *B*=1), and *α*_2_=*α*_1_+π/2, we can simplify Eq. S6 as

|  | $I_{2}={\frac{I_{0}}{4}\cos}^{2} \left( 2\theta-2\alpha_{1}-\pi/2 \right)$. | (S7) |
| --- | --- | --- |

Obviously, because of the orientation degeneracy of the mathematical function $\cos^{2} 2\theta$, there are four orientation angle options to produce equal transmitted intensity, as listed in Tab. S1.

**Case two:** if the nanobrick acts as an ideal half-wave plate (i.e., *A*=1 and *B*=-1) and *α*_2_=*α*_1_+π/2, we can simplify Eq. S6 as

|  | $I_{2}=I_{0}\cos^{2} \left( 2\theta-2\alpha_{1}-\pi/2 \right)$. | (S8) |
| --- | --- | --- |

There are also four orientation angle options to produce equal optical intensity of transmitted light, as listed in Tab. S1.

**Case three:** now we consider a more general condition, if the nanobrick is a general anisotropic scatterer (*A*≠*B*) and *α*_2_=*α*_1_+π/2, we can simplify Eq. S6 as

|  | $I_{2}=\left( \frac{A-B}{2} \right)^{2}\cos^{2} \left( 2\theta-2\alpha_{1}-\pi/2 \right)$. | (S9) |
| --- | --- | --- |

Eq. S9 indicates that our proposed method is not limited to a linear polarizer (case one) or a half-wave plate (case two), and any birefringent nanostructure (*A*≠*B*) can achieve the desired transmitted intensity by rotating the polarization direction of the anisotropic nanostructure.

In addition, when an incident circularly polarized (CP) beam passes through a nanostructure, the Jones vector of transmitted beam can be expressed as

|  | $J_{3}=\left[ \begin{matrix} \cos\theta& -\sin\theta\\ \sin\theta& \cos\theta\end{matrix} \right]\left[ \begin{matrix} A & 0 \\ 0 & B \end{matrix} \right]\left[ \begin{matrix} \cos\theta& \sin\theta\\ -\sin\theta& \cos\theta\end{matrix} \right]\left[ \begin{matrix} 1 \\ \pm i \end{matrix} \right]=\frac{A+B}{2}\left[ \begin{matrix} 1 \\ \pm i \end{matrix} \right]+\frac{A-B}{2}e^{\pm2\theta i}\left[ \begin{matrix} 1 \\ \mp i \end{matrix} \right]$. | (S10) |
| --- | --- | --- |

It can be concluded that the transmitted beam of birefringent nanostructure must include one part with opposite handedness as the incident CP light possessing the phase delay of ±2*θ*. Therefore, combined with the one-to-many mapping between the intensity of transmitted light and rotation angles of the nanostructures, each nanostructure can provide two or four phase delay options, which offers an extra freedom to design phase-only element.

**Table S1 | Summary of the one-to-many mapping scheme.**


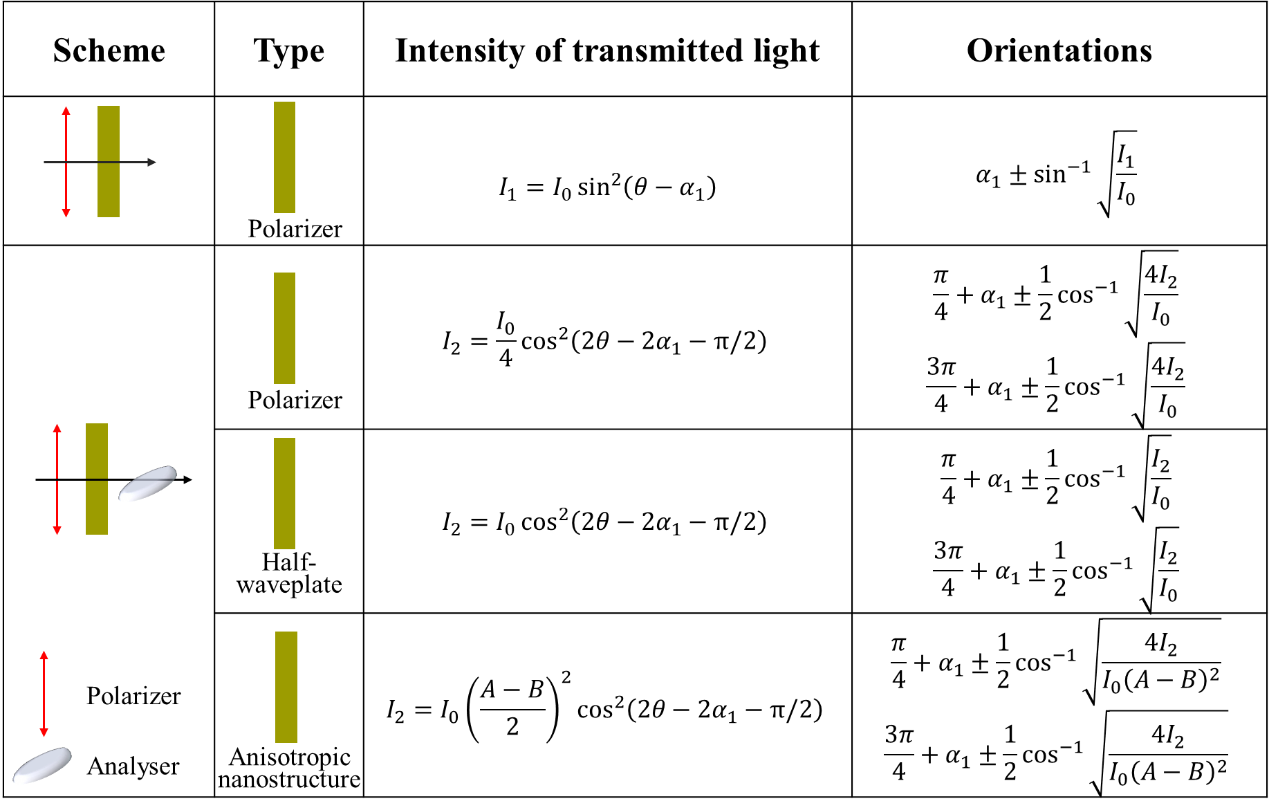


## 2. Simulated annealing algorithm for designing Malus metasurfaces

The flowchart of the simulated annealing algorithm is shown in Fig. S1, which is utilized for encoding an independent holographic image into a greyscale pattern. The simulated annealing algorithm is an iterative optimization algorithm. It starts from an initial phase $\varphi$, which is calculated according to the greyscale distribution of the target greyscale pattern and is exactly twice the orientation angle. Then, a new phase $\varphi_{new}$ is randomly generated at some predefined ranges. Based on the Metropolis acceptance criterion ^[1]^, the candidate phase $\varphi_{new}$ is accepted as the current solution based on the customized search mechanism, that is,$Cost\left( \left| FFT\left( e^{{i\varphi}_{new}} \right) \right|^{2}\text{, }I_{0} \right)<Cost\left( \left| FFT\left( e^{i\varphi} \right) \right|^{2}\text{, }I_{0} \right)$ or $e^{(\Delta I\text{-}{\Delta I}_{new})/t_{k}}>rand(0,1)$, where $t_{k}$ is the temperature of the $k$-th iteration and FFT represents the Fourier transform. When some stopping criterions are satisfied (*e.g.,* a pre-specified maximum number of iterations has been executed or a satisfying solution has been found), we can get an optimal phase distribution that is used to design the Malus metasurfaces. As a result, the designed Malus metasurfaces enable the generation of a greyscale pattern in the near field and the projection of an independent holographic image in the far field.


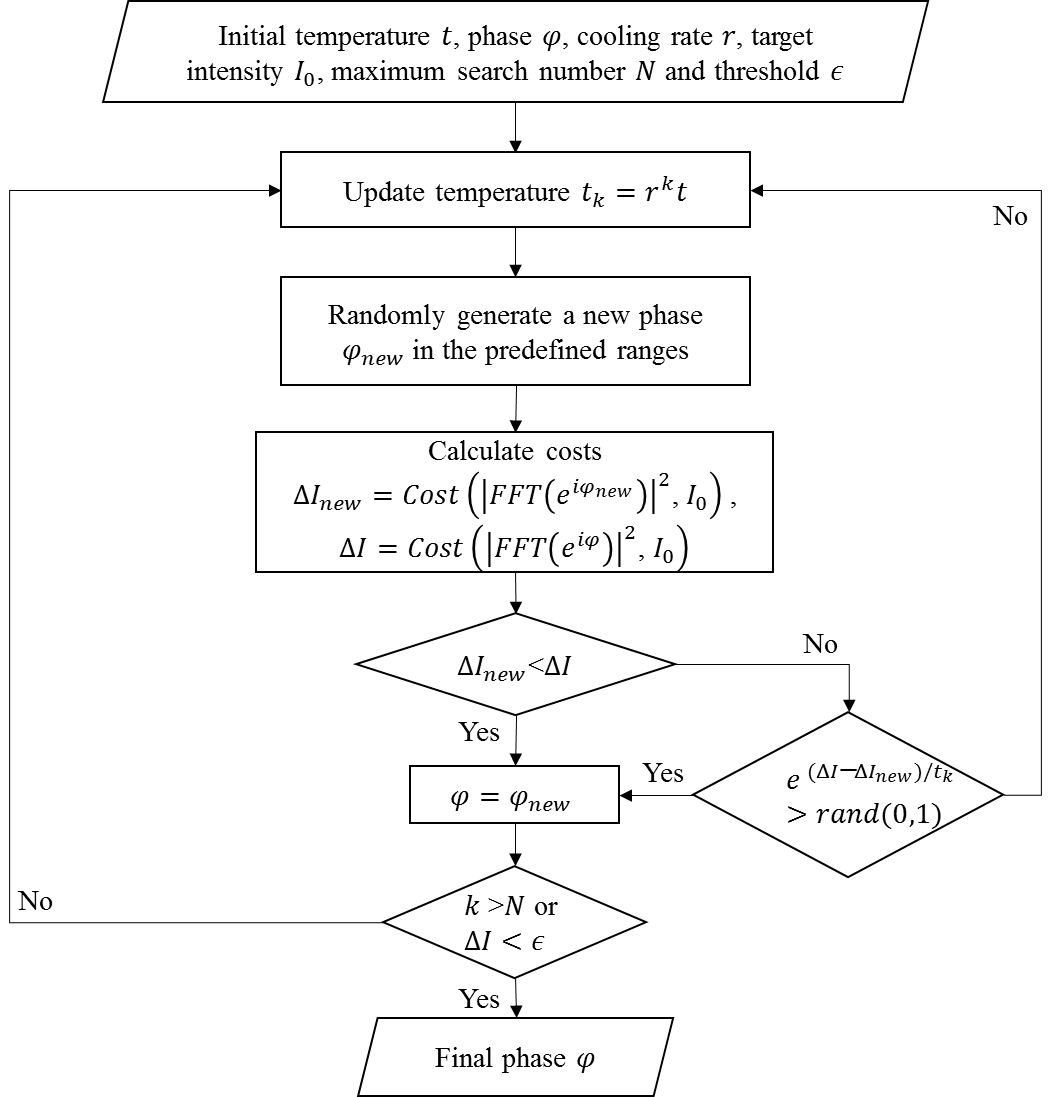


**Figure S1 |** Flowchart of the simulated annealing algorithm for encoding an independent holographic image into a greyscale pattern. $t_{k}$ is the temperature of the $k$-th iteration, FFT represents the Fourier transform, and rand (0,1) indicates a random number in the interval [0,1] can be generated.

The basic configuration of the computer that we use to optimize the final orientation distribution of Malus metasurface is shown in Tab. S2. For the one-to-two mapping scheme, the calculating time is about 70 hours. While for the one-to-four mapping scheme, the time consuming of optimization (about 40 hours) is much shorter. This is because there are more orientation candidates of the nanobricks in the one-to-four mapping scheme, which makes the process of simulated annealing converge quicker and thus less calculating time is required.

**Table S2 | The basic configuration of the computer**

| CPU | Intel Xeon E5-2680 v3*2 |
| --- | --- |
| GPU | NVIDIA Quadra K620 |
| RAM | 192GB |
| OS | Windows 7 |

## 3. Scanning electron microscopy image


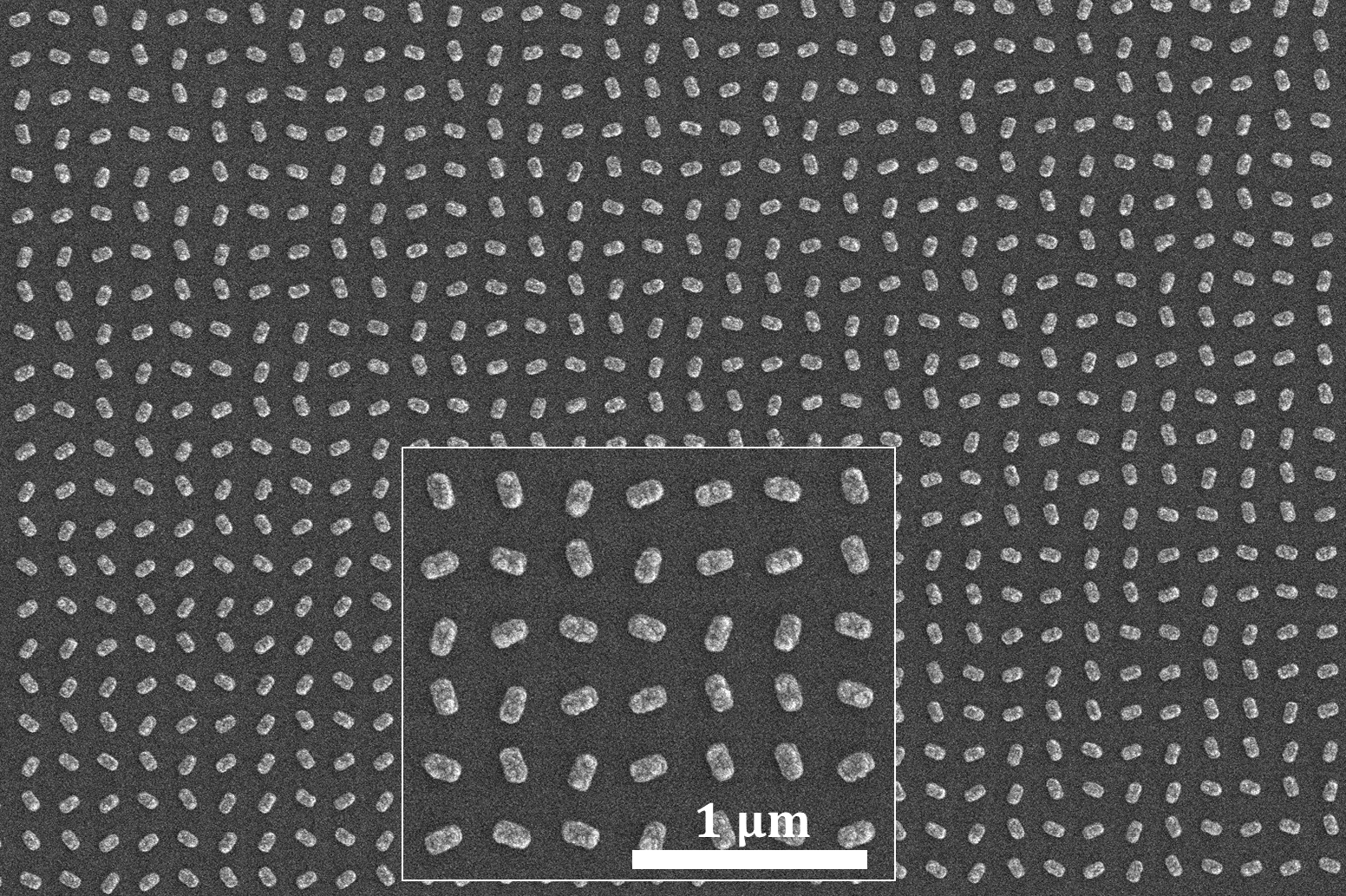


**Figure S2 |** Scanning electron microscopy (SEM) image of sample C (partial view). Before SEM image was taken, the sample was coated with gold using an ion sputter. The scale bar is 1 μm.

## Efficiency calculation and measurement of Malus metasurfaces

For a meta-hologram, we define the diffraction efficiency as the diffraction efficiency of an ideal hologram multiplied by the cross-polarization conversion efficiency of nanostructures.

As shown in Eq. S10, the part with opposite handedness as the incident CP light contributes to the desired holographic image, so the cross-polarization conversion efficiency of nanostructures is defined as $\left| {(\frac{A-B}{2})}^{2} \right|$. In this paper, we employed silver nanobrick arrays (the geometry dimensions are 300 nm in cell size, 160 nm in length, 80 nm in width and 70 nm in height) to form the Malus metasurface. As shown in Fig. S3, **the simulated cross-polarization conversion efficiency is 22.8% at the wavelength of 633 nm.**


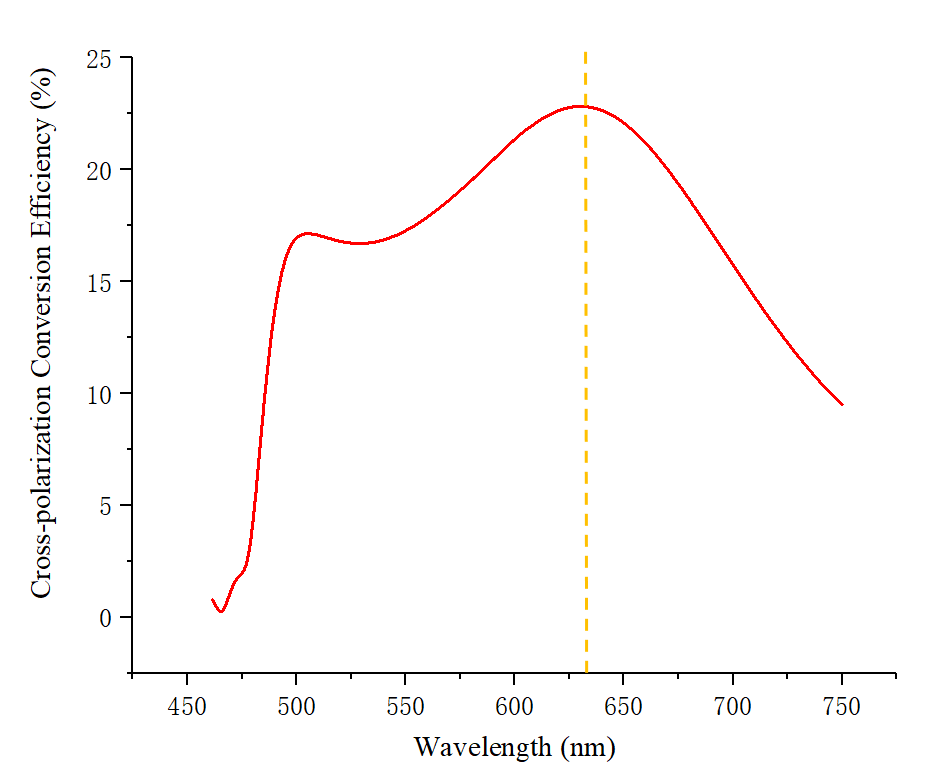


**Figure S3 |** Cross-polarization conversion efficiency of nanobricks acting as polarizers.

Based on the grating diffraction theory^[2]^, the diffraction efficiency of a four-step phase-only optical element is 81%. Therefore, for our designed the four-step Fourier meta-hologram, the theoretical efficiency is (ideally) 22.8%×81%=18.47%.

The optical efficiency measurement setup for the Fourier meta-holograms is shown in Fig. S4. The polarization state of an incident beam from a He-Ne laser was converted into circularly polarization after passing through a linear polarizer (LP) and a quarter-wave plate (QWP). Then, the metasurface sample was illuminated by the CP light after passing through an iris. We used two identical condenser lenses with high numerical aperture (N.A. = 0.6) to collect and focus the diffracted light for the measurement by using an optical power meter (Thorlabs PM100D). The beam power was measured at two points in the light path (i.e., Point 1 and Point 2), as shown in Fig. S4. The measured efficiency is defined as the ratio between the optical power projected into the image region (measured at Point 2) and the input optical power (measured at Point 1).


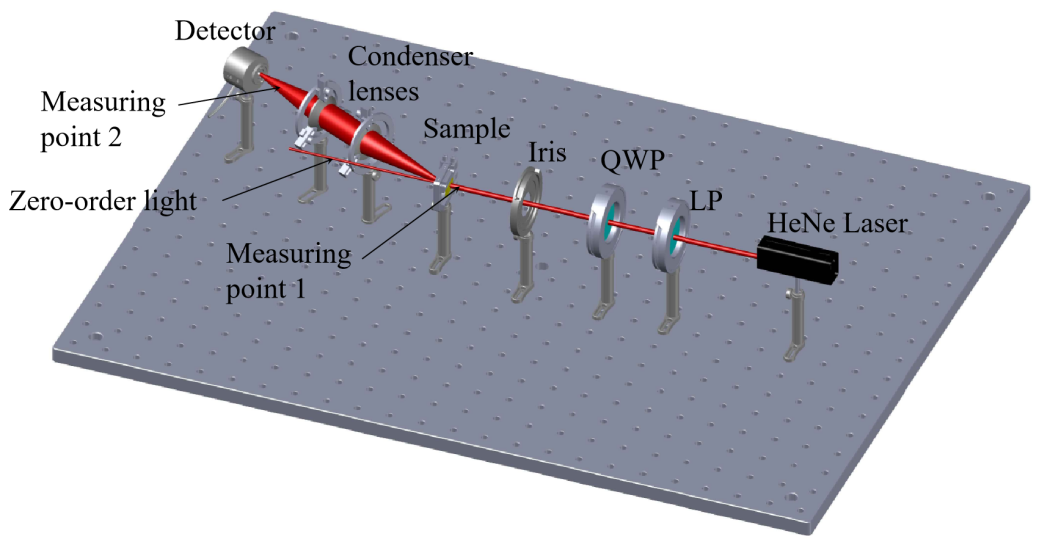


**Figure S4 |** Illustration of the optical efficiency measurement setup for the Fourier meta-holograms.

For our designed four-step Fourier meta-hologram, the measured efficiency is 7% at the operating wavelength of 633 nm. We note that the efficiency is lower than the theoretical efficiency (18.47%). Such an efficiency might be attributed to several reasons. First, the diffraction efficiency is hard to be fully optimized that makes the entire incident beam with the phase delay contributes to the reconstruction of the holographic image. Second, the fabrication errors may cause the inhomogeneous reflectivity of nanobrick arrays and greatly degrade the diffractive performance of the phase-only hologram we designed. Furthermore, the geometric size of the sample is small (300 μm), and the light spot is relatively large, which would also lead to efficiency reduction. It should be noted that although the measured efficiency is not very high compared with the previously reported metasurface work, it arises from technical issues rather than a theoretical limit in principle. Such a relatively low value could be improved by applying more precise fabrication procedures, reducing the coverage angles of the holographic image, using low-loss dielectric materials (such as TiO2) and employing half-wave plate nanostructures.

For the greyscale pattern, the efficiency is difficult to measure since it is recorded right at the sample surface. In addition, as the greyscale pattern is generated by intensity modulation, the transmission efficiency depends on the greyscale distribution of the target pattern in the near field design.

## 5. Experimental results of Malus metasurfaces working in reflection

As shown in Fig. S5, the reflection greyscale patterns and holographic images are captured with the similar experimental setup demonstrated in the main text. The first and second rows of Fig. S5 show the experimental results of samples A-C in the near and far fields, respectively. The third and fourth rows show the experimental results of samples D-F in the near and far fields, respectively.


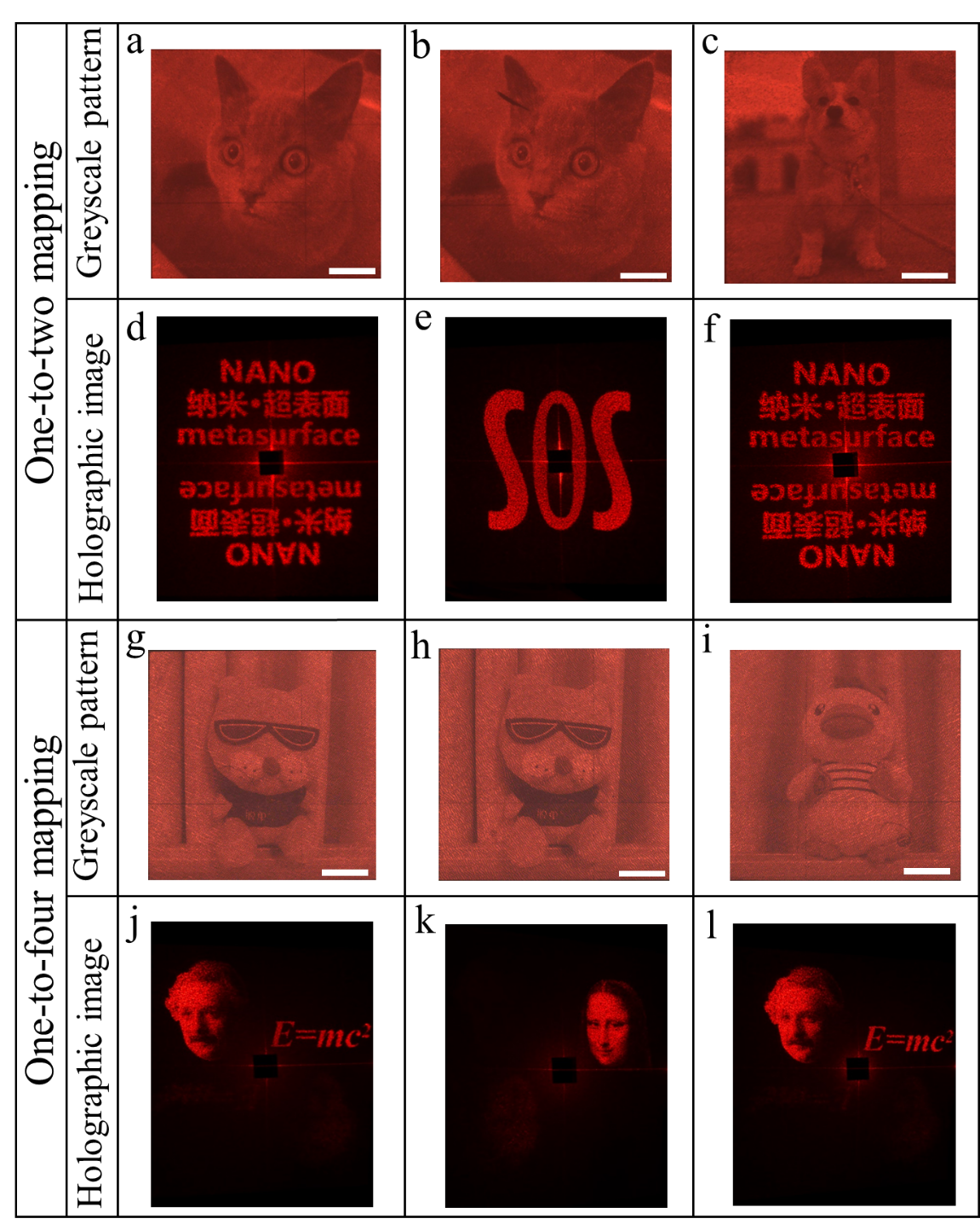


**Figure S5 | Experimental results of samples A-F working in reflection.** (a-f) are experimental images for one-to-two mapping and (g-l) are for one-to-four mapping. First row **(a-c)**: the greyscale patterns of samples A-C under the illumination of LP light. Second row **(d-f)**: the holographic images of samples A-C under CP light illumination. Third row **(g-i)**: the greyscale patterns of samples D-F under the illumination of LP light. Last row **(j-l)**: the holographic images of metasurfaces based on one-to-four mapping under CP light illumination. All samples are designed with dimensions of 150 × 150 μm^2^ and all measurements are conducted at an operating wavelength of 633 nm. The scale bar is 30 μm.

## 6. Broadband response of the nanobrick polarizer based Malus metasurfaces

To investigate the broadband response of metasurfaces based on one-to-many mapping in the near field, we acquire the greyscale patterns under illumination by a quartz halogen lamp using an optical microscope, as shown in Fig. S6. The greyscale patterns obtained in both reflection and transmission show clear visual effects. Additionally, due to the difference of the reflection and transmission spectra, samples A-F exhibit different colours in two different working modes.


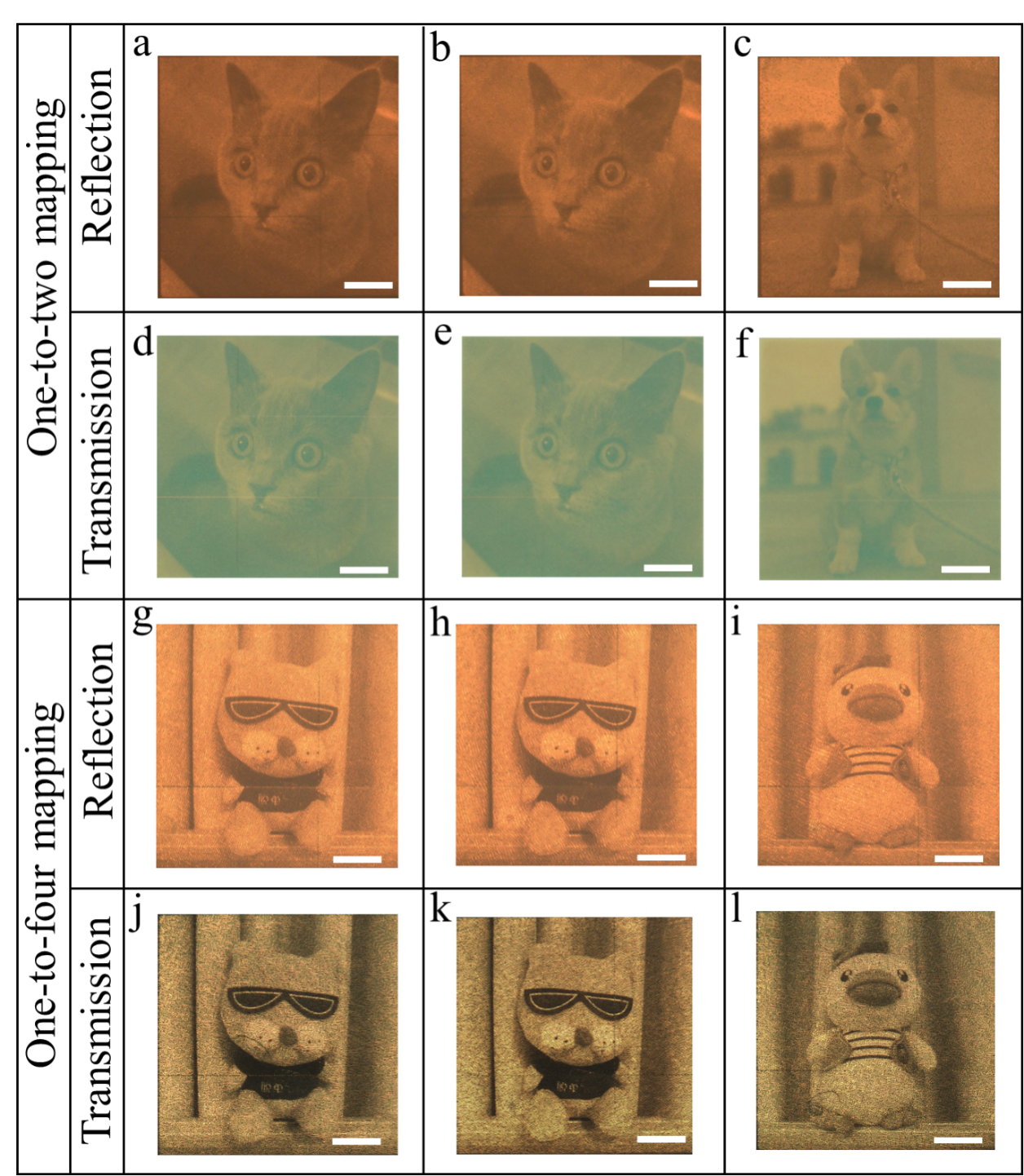


**Figure S6 | Experimental greyscale patterns observed in the near field.** First and second rows **(a-f)**: the greyscale patterns of samples A-C obtained in reflection and transmission. Third and last rows **(g-l)**: the greyscale patterns of samples D-F obtained in reflection and transmission. All experimental results are obtained under illumination by a quartz halogen lamp using an optical microscope. All samples are designed with dimensions of 150 × 150 μm^2^. The scale bar is 30 μm.

To further investigate the broadband response of Malus metasurfaces in the far field, we used a supercontinuum laser source (YSL SC-pro) in the range from 480 to 680 nm in steps of 40 nm to illuminate samples A and D as examples. All the holographic images possess high fidelity, as shown in Fig. S7.


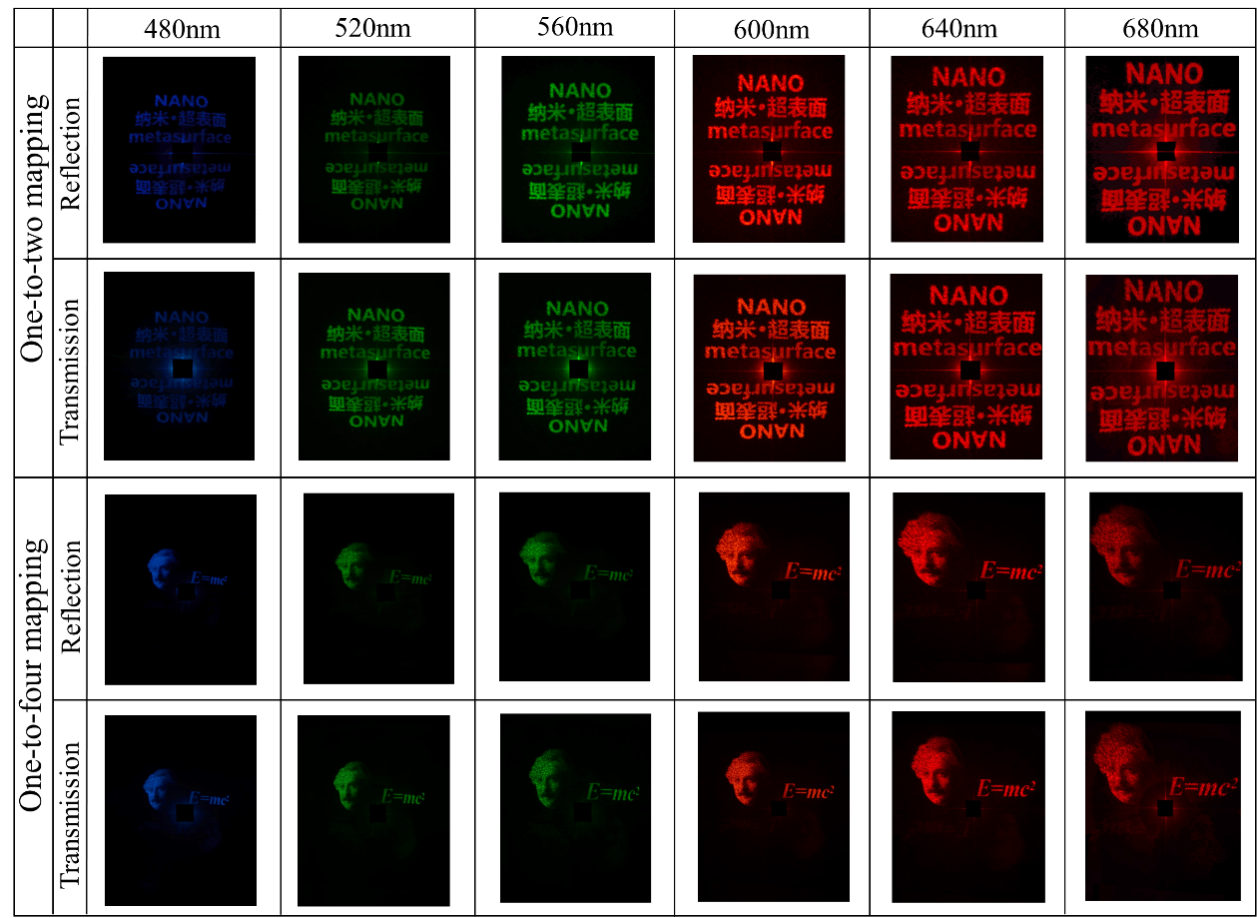


**Figure S7 | Holographic images generated by illuminating samples A and D with a supercontinuum laser source in the range from 480 to 680 nm in steps of 40 nm.** The first and second rows show the experimental results of sample A. The third and the fourth rows show the experimental results of sample D.

**7. Simulation results for four polarization directions of incident LP light and the analyser**

To further understand the difference of conventional one-to-one and our proposed one-to-many mapping schemes, we acquire the simulation results for four typical polarization directions of incident LP light and the analyser, as shown in Fig. S8. The first and second rows show the simulation results of the one-to-many mapping schemes, which have good consistency with the experimental results demonstrated in the main text. According to Eqs. S4 and S7, we design two metasurfaces based on one-to-one mapping by using target patterns of samples A and D, in which the orientation angles of all nanobrick arrays are limited to the interval $\left[ 0,\frac{\pi}{2} \right]$ or $\left[ 0,\frac{\pi}{4} \right]$. As shown in the third and fourth rows of Fig. S8, when the polarization orientation angle of incident LP light is rotated from the designed angle to others, the target greyscale patterns cannot be hidden.


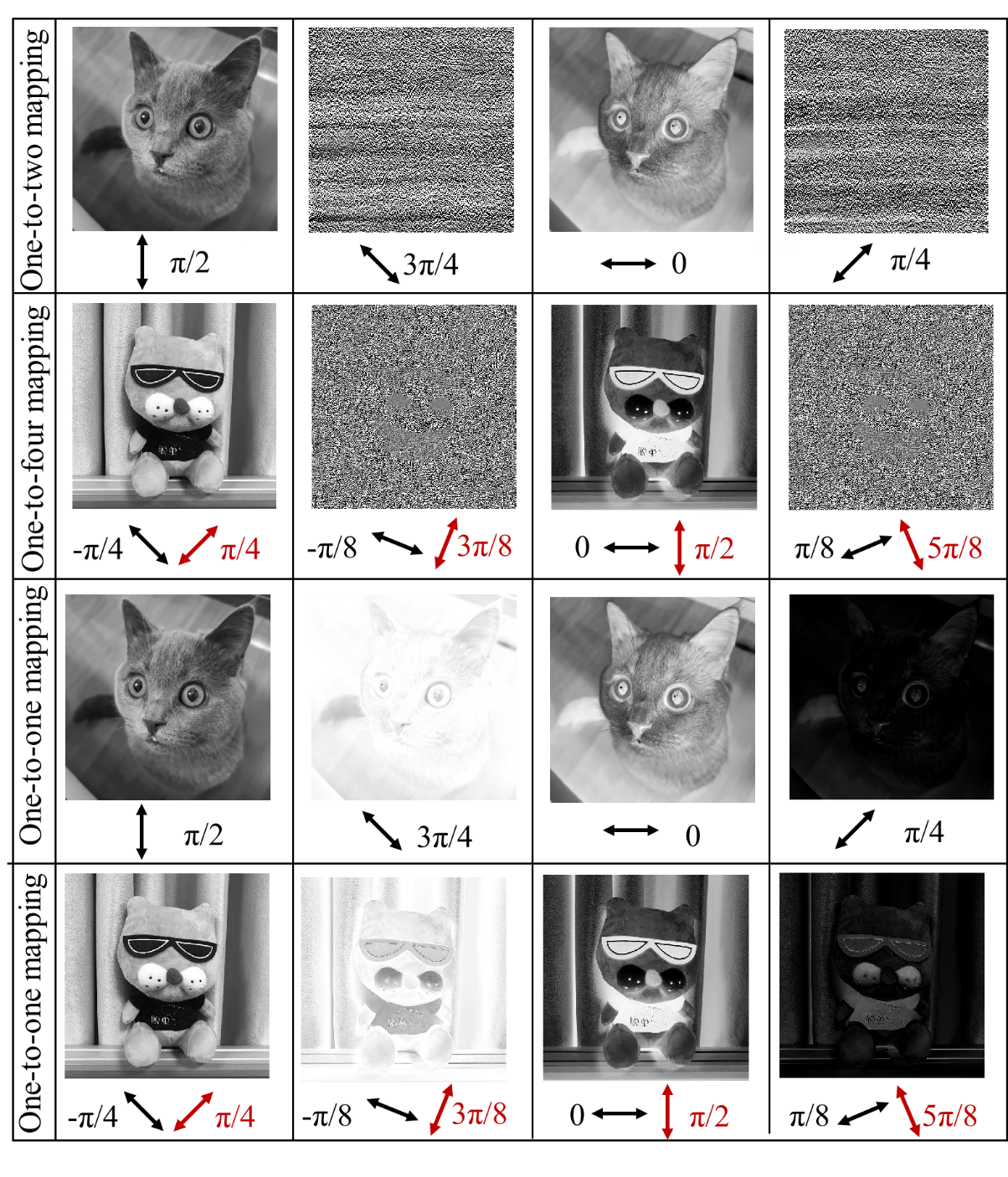


**Figure S8 | Simulation results for** **four polarization directions of incident LP light and the analyser.** The first and second rows show the simulation results of metasurfaces with one-to-many mapping schemes. The third and the fourth rows show the simulation results of metasurfaces with conventional one-to-one mapping schemes.

**8. One-to-eight mapping scheme based on a bilayer metasurface**

Our proposed method can be further extended to one-to-eight mapping scheme by introducing the bilayer metasurfaces, but at the cost of increasing fabrication complexity.

For example, we can design the bilayer nanostructures consisting of a nanobrick polarizer (nanobrick short axis is transmission axis) and a nanobrick half-wave plate to achieve the one-to-eight mapping, as shown in Fig. S9. When the incident *x*-axis polarized light with an intensity of $I_{0}$ passes through the bilayer nanostructures in the forward or backward direction, the intensity of the transmitted light can be expressed as

|  | $I_{t12}=I_{0}\sin^{2} \theta_{1},$ | (S11) |
| --- | --- | --- |
|  | $I_{t21}=I_{0}\sin^{2} {(2\theta}_{2}-\theta_{1}),$ | (S12) |

where *θ*_1_ and *θ*_2_ are orientations of the nanobrick polarizer and nanobrick half-wave plate, respectively.


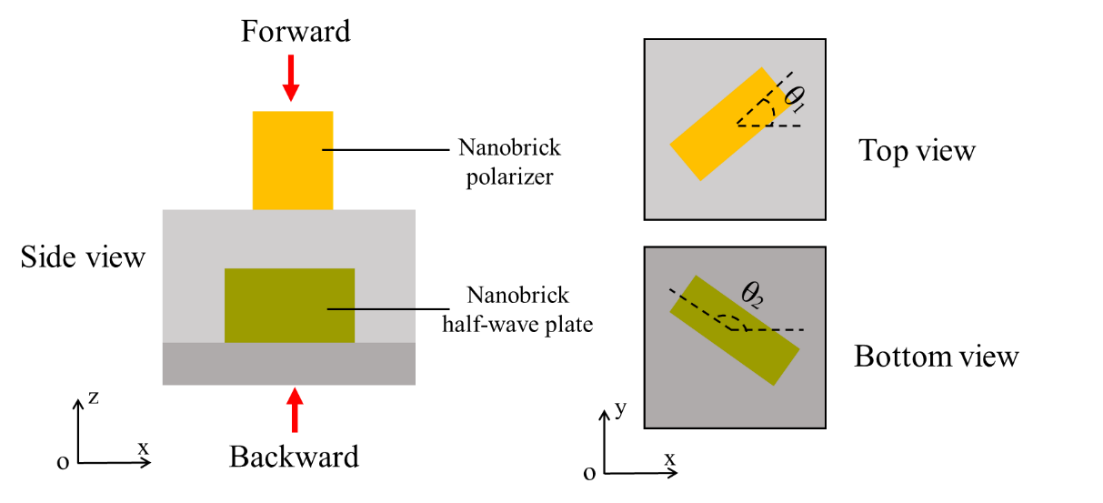


**Figure S9 | Side view, top view and bottom view of the bilayer nanostructures.**

As a result, when the intensity of incident light is given, two continuous greyscale images can be formed at the upper and lower surfaces of the bilayer metasurfaces by configuring $\theta_{1}$ and $\theta_{2}$. More importantly, inspired by the orientation degeneracy we propose, there are eight combinations of $\theta_{1}$ and $\theta_{2}$ that can generate fixed intensity $I_{t12}$ and $I_{t21}$, which corresponds to eight different geometric phase delays ${2\theta}_{1}-{2\theta}_{2}$. As an example, to simultaneously meet $I_{t12}$=0.75 and $I_{t21}$=0.5, we have eight candidates of ${2\theta}_{1}-{2\theta}_{2}$ that correspond to eight different geometric phase delays, as shown in Table S3.

**Table S3 | An example of one-to-eight mapping**


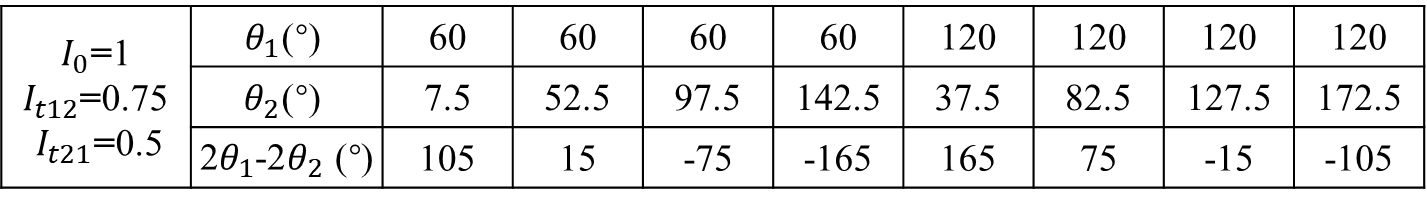


In summary, our proposed method can be further extended to one-to-eight mapping. By stacking more layers of nanostructures in space, the method we propose is expected to achieve one-to-higher mapping.

## Reference

1. Metropolis, N. *et al*. Teller, E. Equation of state calculations by fast computing machines. *The journal of chemical physics.* **21**, 1087-1092(1953).
2. Cox J. *et al*. Diffraction efficiency of binary optical elements. *Proceedings of SPIE - The International Society for Optical Engineering,* **1211**, 116-124 (1990).
